# Research Mentoring Plan Involving North-South Collaborations

**DOI:** 10.1200/JGO.19.00176

**Published:** 2019-08-21

**Authors:** Thenjiwe Emily Major, Bramwell Koyabe, Mohan Narasimhamurthy, Oathokwa Nkomazana, Harvey M. Friedman

**Affiliations:** ^1^ University of Botswana, Gaborone, Botswana; ^2^ University of Pennsylvania, Philadelphia, PA

Globally, the prevalence of noncommunicable diseases (NCDs), such as cancer and cardiovascular disease, is on the rise, placing burdens on economic growth and health systems in resource-limited settings.^1^ In sub-Saharan Africa, the situation is exacerbated by the increasing prevalence of HIV and the associated infectious diseases. In this article, we describe the impact of a Mentoring Core funded through a grant from the US National Cancer Institute of the National Institutes of Health (NIH) that focuses on North-South collaborations to build research capacity in Botswana, Africa, in topics related to HIV and cancer. We describe the goals of the mentoring program, two research projects awarded through pilot grant funding, and discuss how individual junior faculty members at the University of Botswana benefited from the mentorship and collaborations that helped them gain research skills in HIV and cancer.

Morbidity and mortality from NCDs account for approximately 31% of all deaths in Botswana, where cancer, cardiovascular disease, and diabetes are among the most common NCDs.^2^ In recent decades, the magnitude of the NCD burden in sub-Saharan Africa has been overshadowed by HIV/AIDs and the re-emergence of tuberculosis. Sub-Saharan Africa still has the highest HIV prevalence rates in the world. HIV infection is associated with higher incidence and more rapid progression of human papillomavirus (HPV)-associated malignancies, such as head and neck, penile, anal, and cervical cancers.^3^ There is an urgent need for applied health research that will lead to policies and action plans to address the increasing burden of NCDs and infectious diseases on the health system.

In September 2014, the National Cancer Institute of the NIH funded an interdisciplinary research consortium between the University of Botswana and the University of Pennsylvania. The focus of the research consortium is on studies related to cervical and other cancers in individuals in Botswana who have HIV infection. The grant provided funding for a Mentoring and Career Development Core aimed at building research capacity of junior faculty at the University of Botswana without requiring them to spend extended time training at a US university.

The Core directors developed an educational program on grant writing and research methodology and established a mentored pilot grant program for research proposals on topics related to cancer in individuals in Botswana who have HIV infection. A 2-day course is held annually on the University of Botswana campus with instructors from the University of Botswana and the University of Pennsylvania. Approximately 70 students and junior faculty from the University of Botswana attend each year. The typical curriculum involves a half day of lectures on topics including epidemiology of cancer in people with HIV in Botswana; updates on treatments available for patients in Botswana with HIV who also have cancer; information on how a biostatistician can assist with protocol development; institutional review board submission guidelines; preparation of a research protocol; developing a theoretical framework and assessment section of a grant proposal; and information on how to write a pilot grant.

At the conclusion of the lectures, participants interested in submitting an application as a principle investigator (PI) are asked to present their research ideas to the other participants. Generally, six to 10 individuals express interest in submitting a pilot grant application as a PI. The remaining participants are asked to align themselves with a PI on a topic of interest to them. The remaining time on day 1 and the morning of day 2 are spent with participants gathered in small groups headed by the PIs as they develop the specific aims of their proposals. Senior research faculty from the University of Botswana and University of Pennsylvania circulate among the groups assisting the PIs and the co-PIs in developing their specific aims. The last several hours of day 2 are spent with each PI presenting the specific aims and hypotheses to the entire class, with time set aside for discussion.

The PIs are asked to continue to develop their project over the next several weeks and to submit their specific aims to the course directors within 1 month. The course directors then assign scientific mentors to each PI on the basis of the topic of their application. The intent is for the mentor to assist the PI develop their pilot grant application over the next 3 months. The final application includes résumés of the PI and co-PIs (up to three pages each), an Abstract of no more than 30 lines, specific aims (one-half page) and research strategy (2.5 pages) that include sections on significance, innovation, and approach. A mentoring plan (one-half page) completes the application.

Generally, four to six applications are submitted annually. Two scientists from the University of Botswana and two from the University of Pennsylvania review each application. Written feedback is provided to the PIs using an NIH scoring system on a scale of 1 to 9, where 1 represents the best score. Scoring criteria include the significance of the research, the approach taken to address the problem, innovation, and quality of the investigator. Two pilot grants are funded annually for 125,000 Pula (approximately US$12,500). The scientific mentors continue to work with the PIs of applications that were not successful to improve the application for future submissions. For the funded applications, the scientific mentors advise the PIs as they prepare submissions for review by the Human Subjects committees in Botswana and at the University of Pennsylvania, develop their study protocol, implement their study, and prepare a manuscript for publication. University of Botswana officials assist the PIs in handling administrative and financial obligations according to local regulations. In the next section, we provide information on the first two funded projects and highlight the impact the funded applications had on the career of the PI.

## Norms and Beliefs Related to Cervical Screening

One of the first projects funded was a case study that evaluated norms and beliefs related to cervical cancer screening in women ages 25 to 49 years in Botswana. The study was performed in two phases. In phase 1, a qualitative, descriptive, and contextual research approach was used to understand attitudes, behavioral intentions, subjective norms, social norms, perceived power, and perceived behavioral control. The case study approach was used to obtain in-depth, first-hand information from women regarding cervical cancer screening. The theoretical frameworks included the theory of reasoned action; its extension, the theory of planned behavior; and social cognitive theory.^4,5^ Focus group discussion and semistructured interviews were administered to women in two villages. The results of this phase 1 study revealed behavioral, normative, and control beliefs concerning cervical cancer screening and a lack of knowledge about cervical cancer that contributed to failure to screen.

Phase 2 of the pilot study used the phase 1 data to design a survey questionnaire according to the theory of planned behavior.^4,5^ The instrument had 48 items on demography and 42 items on cervical screening. Items were categorized in four themes: Intentions, Attitudes, Behavioral Beliefs, Normative and Control Beliefs. A total of 140 participants enrolled in the study. The results showed that Normative Beliefs was a significant contributor to whether women participated in cervical cancer screening. These findings will be used to design a culturally contextually appropriate phase 3 intervention to promote cervical screening among women in Botswana.

Regarding benefits of the mentoring program, the mentee acknowledged the importance of being mentored by skilled and experienced researchers. Mentors offered this PI guidance, particularly in writing the pilot grant proposal. The mentee stated that the mentored pilot grant program improved her grant writing skills and provided experience preparing a grant to meet the NIH standards. She gained experience and knowledge in designing and implementing studies in a culturally sensitive fashion. The mentoring program helped this PI establish a broader network of scientific collaborators, including international collaborators. The research skills acquired will improve her ability to serve as a mentor to undergraduate trainees and junior faculty. She attributed her joy of being a mentor to the good mentoring she received from the project.

Because of the pilot grant funding, the mentee has been invited to present her research at national and international meetings. She has been successful in writing scholarly papers submitted to reputable journals, including one that has been published,^6^ another that was published as an abstract,^7^ and a third manuscript that is under review. She is currently in the process of applying for external, peer-reviewed funding for the phase 3 intervention, and was recently promoted to Senior Lecturer, based in part on her research productivity.

## Identification of Differential Expression of Host Genes in Cervical Cancer

Not all women chronically infected with oncogenic HPV serotypes progress to cervical cancer, which suggests other factors are important in the causal pathway of cancer development. A goal of this research project is to identify epigenetic changes associated with cervical cancer progression in women with and without HIV infection in Botswana. Another goal of the study is to help develop the technology needed for future studies that evaluate the mechanisms of tumor progression in people with HPV. The study is in progress and involves three phases. Phase 1 was to standardize the polymerase chain reaction techniques used to determine HPV genotypes in paraffin-embedded tissues. The study has now been published.^8^ The second phase was to identify the prevalence of HPV genotypes among the study population. A manuscript is under review. The third phase is ongoing and evaluates the methylation status of host genes. The goal of the third phase is to evaluate the potential role of HIV and other cofactors in epigenetic mechanism of carcinogenesis.

The pilot grant program has brought together a highly motivated team of researchers at the University of Botswana that is helping build research capacity in Botswana. The mentoring program helped the PI gain insight into research methodology and helped him establish collaborations with many researchers at the University of Botswana and the University of Pennsylvania. The mentee interacts with his mentors monthly, which has helped him acquire the skills required for grant writing, data acquisition, and protocol development. The grant writing skills acquired from this project helped him successfully apply for three additional grants funded by the University of Botswana. He has learned critical research skills in a challenging research environment that has few senior, experienced researchers. In addition, the program has helped him develop his own mentoring skills. He is currently serving as mentor to two Master of Philosophy students at the University of Botswana.

The pilot grant funding facilitated the pilot grant PI’s participation at international meetings sponsored by the United States and Canadian Academy of Pathologists and the European Society of Pathologists. He has written one published paper^8^ and another that is under review. His research productivity contributed to his recent promotion to Senior Lecturer at the University of Botswana.

In conclusion, HIV and cancer are leading causes of death in Botswana. Therefore, research on HIV and cancer is an important focus for Botswana. Although the government of Botswana provides antiretroviral therapy to all people infected with HIV, these drugs do not prevent progression of some oncogenic virus infections. Local researchers, including faculty at the major universities in Botswana, are highly motivated to address these issues. The Mentoring and Career Development Core helps faculty perform research in a culturally and contextually appropriate manner without requiring them to train over prolonged periods abroad.
